# An international survey of medical licensing requirements for immigrating physicians, focusing on communication evaluation

**DOI:** 10.5116/ijme.5690.ef62

**Published:** 2016-02-06

**Authors:** Amy Gillis, Rebecca Weedle, Marie Morris, Paul Ridgway

**Affiliations:** 1 Department of Surgery, Tallaght Hospital, Tallaght, Dublin 24, Ireland; 2 Education Division, School of Medicine, Trinity College, Dublin, Ireland

**Keywords:** Communication, standardisation, international, assessment

## Abstract

**Objectives:**

To identify current entry requirements set by international medical licensing bodies for immigrating physicians, focusing on postgraduate level communication skills, clinical and technical skill assessments.

**Methods:**

A standardised, author developed survey was administered to a selection of national, state and provincial licensing institutions across 6 continents. Representative institutions were selected from the most populated regions of each continent. Surveys were administered by email and telephone. The information was also searched by website review. Website information alone was used if no response was received by the targeted institution after 2 phone/2 email attempts. Statistical analysis of the non-parametric data was conducted using SPSS (v.21).

**Results:**

Thirty-seven licensing bodies were contacted from 30 countries; verifiable information was available for 29; twenty-six responded to the communication inquiry. Sixty five 65.4% (n=17) surveyed communication skills, 100% involved language proficiency testing; 11.5% tested other forms of communication skills. For clinical and technical skills, 86.2% (n=25) assessed candidates by credential review, 72.4% (n=21) required both credential review and exam and 62.1% (n=18) used country-specific examination. A mentorship period were required by 37.9% (n=11), ranging from 3 months to 1 year. Only 2 countries identified examinations for recertification. No technical/clinical skills nor communication skill evaluation (beyond language proficiency) are routinely assessed at the postgraduate level.

**Conclusions:**

International assessments of migrating physicians are heterogeneous. Communication skills, beyond language proficiency, are not routinely assessed in foreign trained physicians seeking entry. The majority of clinical and technical skills are assessed by credential review only. This study highlights the lack of standardisation of assessment internationally and the need for steps toward a global agreement on training schemes and summative assessment.

## Introduction

The migration of physicians has become a world-wide phenomenon in the current economic climate.  There is a recognized shortage of quality trained health care professionals. International medical graduates make up 23.1-28.3% of the physician workforces of the UK, US, Canada, and Australia.^1^ There is constant movement within countries, from rural to urban areas, and between countries.^2^ The movement of physicians is driven by a number of social factors, namely financial remuneration, educational opportunities and family influences. The movement of physicians creates ethical dilemmas that need to be addressed globally. Each country has its own unique health care system, and the established training systems for medical professionals reflect these differences.  Evaluating readiness for independent practice is complex, requiring the necessary knowledge base, technical and clinical skill acquisition, but examination of the literature reveals no standardisation of accreditation across countries to regulate the quality of physicians entering into the work force, at the point of transition to independent practice.

Navigation through medical licensing has been described as “complicated, time consuming and expensive.”^3^ Over the past twenty years, agencies have been making efforts to internationally standardise the accreditation of healthcare bodies in an effort to address these issues. ^4^

Adjudication of fitness for practice is assessed by numerous methods which include review of credentials, written examinations, examinations by OSCE (Objective Structured Clinical Examination), and mentored practice.

In addition to the necessary knowledge and clinical bases, non-technical skills such as communication are necessary for ensuring fluid and high standards of patient care.

Communication failures are one of the commonest causes of inadvertent patient harm,^5^ are identified as the root cause of 65% of sentinel events^6^ and are factors in up to 80% of errors that result in death or permanent loss of function for patients.^6^

Communication skills are taught and assessed at the undergraduate level, and as noted by Morris et al.^7^, positive attitudes toward communication skills were shown to diminish with progression through the primary medical degree, as the current culture of medicine traditionally “deemphasises the importance of communication skills”.^7^ In the postgraduate setting, and particularly at the point of transition to independent practice, there is little known in the literature as to how applicants are assessed for readiness to practice globally, particularly in non-technical aspects of medical care.

The primary aim of this study is to survey international medical licensing bodies to determine whether communications skills are formally assessed for physicians transitioning to independent practice or applying for licensure in a new country and note the tools used for communication skills assessments.  Secondary outcomes look to identify current entry standards and assessments used by individual medical licensing bodies for knowledge and clinical skills.

## Methods

### Study design

An author developed, standardised survey was developed and administered to national, state and provincial licensing institutions across 6 continents. The standardised survey was composed of 5 questions, with proscribed answers as well as an option to input original answers. Questions centred on communication skills, knowledge base testing, technical and clinical skills evaluation and the tools used in these assessments.

### Sampling methods and selection criteria

The most populated countries of each of the six permanently populated continents were chosen as representatives for the survey. The survey aimed to address the licensing bodies in countries that carried the larger bulk of the world’s population. The representative licensing bodies for the most populated countries were identified by an online search. Licensing bodies were defined as the governing bodies responsible for medical licensing. Countries with non-centralised medical licensing boards were contacted separately with the most populated regions of those countries identified as representative. Specialist licensing institutions (e.g. separate subspecialty licensing surgical bodies) were not specifically targeted.

### Sample size

Thirty-seven licensing bodies were identified from 30 countries based on the selection criteria.

The projected sum population to be represented by this group was 4.298 billion people, approximately 61% of the world’s population (Table 1).

Eleven of these countries were identified as possessing a developing economy based on the World Bank classification for 2013-2014. (Assessed by a GNI (gross national income/capita/year) of <US$11 905/capita/year).^8^

**Table 1:** Percentage of population per continent and countries per continent

Continent	No. countries	Population	No. countries/ continent included in survey	% Population represented from continent	% Countries per continent represented
Africa	54	1,037,524,058	4	34.2	7.4
Asia*	44	4,307,107,875	9	70.3	20.4
North America**	23	544,620,340	7	52.8	30.4
South America	12	400,067,694	2	60.3	16.7
Europe	47	816,426,346	6	32.2	12.8
Oceania	14	35,426,995	2	78.2	14.3

*Russia and the Middle East are included as part of the Asian continent geographically

**Central America and the Caribbean are included as part of the North American continent geographically

### Data collection

The survey was distributed through two attempts at email contact and two attempts with telephone contact. Website information was also searched and all information gained from the survey was verified and supported by web based documents. Where neither telephone nor email contact elicited responses, the gathered website information and available documentation was used to complete the survey. 

### Data analysis

Statistical analysis of frequency data was conducted using SPSS (V.21, Armonk, NY, USA).^9^

### Ethics

Ethical approval was granted by the Faculty Research Ethics Committee at Trinity College Dublin of the University of Dublin. Data was anonymized prior to analysis to preserve the confidentiality of the survey respondents. Participant response is interpreted as implied consent. 

## Results

### Data demographics

Contact was attempted for the thirty-seven licensing bodies identified from 30 countries based on the selection criteria. Verifiable information was available from 29 licensing bodies in 22 countries.  For the remaining eight countries, either no contact information was available or verifiable information could not be collected from appropriate websites.

Of these 29 licencing bodies, 26 responded to the first question, revealing 65.4% (n=17) assessed communication skills specifically, 100% of these involved language testing. Information gathering in the form of history taking was assessed as part of the integrated OSCE exam by 5 licensing institutions, and 11.5% (n=3) tested any other communication forms, e.g. breaking bad news, consent, patient instruction.

### Communication assessment

**Figure 1:**
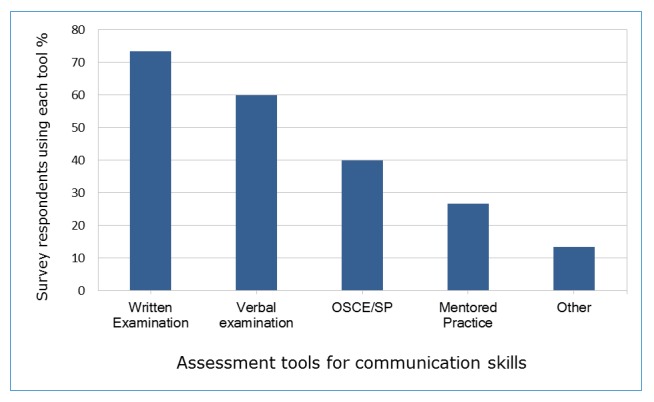
Measurement of communication skills

The methods of communication assessment varied and were often used in tandem. Language proficiency with written and verbal examination was most often assessed by a commercially available exam e.g. TOFEL.  An OSCE was conducted by 35.3% (n=6) to assess language during clinical skill evaluation.  A period of mentored practice was used by 23.5% (n=4) to observe clinical skill and communication. The remaining methods consisted of structured interview settings to assess language skill (Figure 1).

### Knowledge base assessment

**Figure 2:**
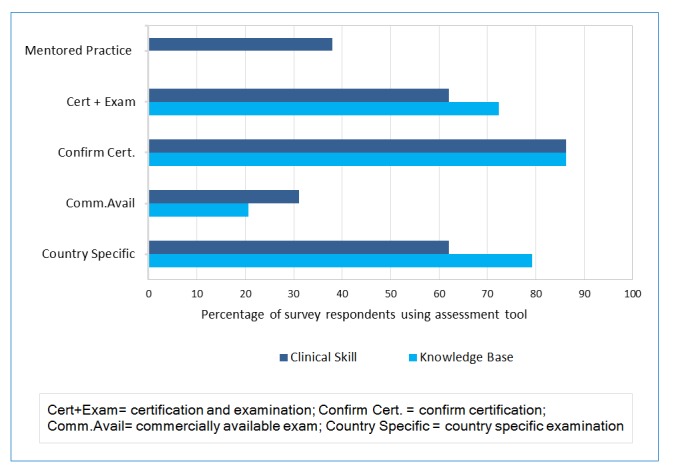
Knowledge base and clinical skill assessment methods

Knowledge based assessment was performed in all regions. 79.3% (n=23) used a country or region specific examination. Less than 1/3 (n=6) used a commercially available exam (most common example was the USMLE (United States Medical Licensing Examination) used within and outside of the United States).  More than 80% (86.2% n=25) used confirmation of certification and references in some capacity, for determining knowledge and 72.4% (n=21) used both review of certification and examination.  Fourteen percent 14% (n=4) used credentialing alone (Figure 2).

### Technical/clinical skill assessment

Technical or clinical skill assessment encompassed any skill set that excluded knowledge based or communication skills assessment. These were predominantly assessed by credential review, 86.2% (n=25), by written exam and confirmation of certification (62.1% n=18) and <40% (n=11; 37.9%) required engaged mentored practice of a variable duration from 3 months to 1 year (Figure 2).

Re-certification was assessed by 2 licensing bodies for practicing physicians at varying points in a career.

## Discussion

This brief survey highlights the hetrogeny of assessment methods that exist from country to country. Heterogeny in assessment can reflect a potential for significant heterogeny in training. As globalisation of medicine continues, it reflects a growing need to establish an agreed collective means of both training and assessment of doctors internationally.

In general, the majority of global applicants are assessed for all aspects of training by a review of current credentials and source country references (86%) in conjunction with a basic language examination.  When addressing communication skill set, this is minimally assessed. No standardised assessment criteria have been identified that exists between countries. At the point of transition to independent practice, language proficiency is the only routinely practiced assessment of communication skill, most following a commercially available exam. Other skills, such as consent or breaking bad news, are rarely examined. If assessed, these skills are evaluated as components of OSCEs, but the extent and nature of the skills assessed as a component of an OSCE station is unstandardized.

For technical or clinical skills assessment, credential review and OSCEs are again the most common form of clinical/technical skill assessment.  The OSCE is most often country specific and is developed to best represent the skills that a particular licensing body has identified as necessary. The evaluation tool used within an OSCE is most often a checklist designed by the evaluating group, assigning value to the specific attributes. Though the OSCE allows a wide range of skills sets to be assessed, OSCEs are subjective. OSCEs have been criticised for the artificiality of the scenarios, not able to accurately mimic real life situations.^10^ Reliability of OSCEs has been questioned due to the variations in internal reliability depending upon number of stations, and test length.^11^ Though country specific examinations allow a tailored assessment, they are open for bias and potential under-assessment of candidates as stations are not standardised, from country to country.

### Mentored practice

The use of mentored practice likely gives the best assessment or overview of a physician’s ability to practice and the capacity to identify any safety issues.  This is a more time consuming process for a licensing body to arrange, and may not be feasible on a scale to accommodate the volume of migration that a number of countries see.  Mentorship occurred in 37% of cases in this survey and was of a variable time frame, from three months to one year.  Though some countries or licensing bodies required mentorship for all applicants, exact criteria as to who qualified for a mentorship in the majority of countries was not clear, and was generally reserved to the discretion of the licensing body after credential review.

### Access

The information gathered here was difficult to access. Trying to identify the most appropriate department or the most responsible licensing body who would best be able to give the required information was not readily evident.  Physicians attempting to migrate may experience difficulty in accessing the necessary information for application. 

### Impact for medical educationalists

The impact of non-standardisation for the medical community is evident. Governing bodies in medical education strive for the highest standard of teaching and assessment to guarantee fitness to practice of graduates.  This is important for medical educators in general and medical licensing bodies in particular for domestic graduates and also foreign graduates that seek to work in a new medical system.  The current status of assessment, as demonstrated by this survey, evaluates foreign applicant’s fitness for practice predominantly by credential review, relying on the inherent expectation that all medical systems train their trainees in the same manner and that assessment is equivalent. This is not the case, neither in current practice nor in how different education systems are perceived by each other.^12^This survey highlights the lack of necessary cohesion in training bodies surrounding the world, and the areas for potential standardisation development.

### Limitations

This paper carries a number of limitations. The survey was author developed and unvalidated, containing only 5 questions, and does not address specifics of how examinations are developed, particularly individual OSCE components, and the emphasis placed on communications skills in the specific OSCE.  Due to some difficulty in obtaining information, and the nature of surveys, the most appropriate and informed person may not have been the person who completed the survey.

## Conclusions

Overall there is a discrepancy on requirements for certification for independent practice between countries and between licensing bodies within the same country. In communication skills there is no standardised assessment beyond language proficiency. Clinical and knowledge based exams are heterogeneous, and country specific, though the majority follow similar formats of written examination and general OSCE clinical examination. For medical educators, the next step is to attempt to develop a collective, internationally recognised standard of what constitutes the competent medical physician, and to validate methods of assessment of these tools that are transparent and objective.

### Conflict of Interest

The authors declare that they have no conflict of interest.
